# Supplementary material for: A street corner education: Stop and search, trust, and gendered norms among adolescent males
Source: PLoS One. 2022 Dec 27;17(12):e0279505. doi: 10.1371/journal.pone.0279505 (PMC9794084; doi:10.1371/journal.pone.0279505)
Supplement: Attachment — Submitted filename: Response to Reviewers.docx [file pone.0279505.s002.docx]

**A street corner education: Stop and search, trust, and gendered norms among adolescent males**

We thank the reviewers for their positive and very useful comments on the previous draft of our paper. We are particularly glad the reviewers saw the potential of the paper, as a primarily theoretical piece bolstered by empirical evidence, to “pave a clear pathway for future research and data collection to assess causal effects” ( Reviewer 1). We address each of the points the reviwers raise below, but as a general point would note that in response to their comments we have tried to simplify and clarify the text, use more appropriate caveating around causality, and strengthened some of the other literature covered.

We should also note that we have slightly amended the title of the paper, to *A street corner education: Stop and search, trust, and gendered norms among adolescent males.* We think this better reflects the content. While reviewing the paper we also spotted that the sample sizes for some analyses were wrong (i.e. cases had been inappropriately excluded). This has now been corrected – the only substantive effect was to shift the significance level for one path in the SEM in Figure 2 (Trust 🡪 Norms 2) from ‘.05’ to ‘.10’.

**Reviewer #1**

• First, although the data for this paper were collected in the UK, there is some work based on youth and young adults in US cities that are relevant for the paper – specifically, Monica Bell’s (2017, Yale Law Journal) work on procedural injustice is critical to cite. In addition, Amanda Geller (2021, American Journal of Public Health) provides population level prevalence of police contact among adolescents, and Geller and Fagan (2019, Russell Sage Foundation journal) provide an examination of police contact and legal socialization. Additional work based on the Fragile Families and Child Wellbeing Study may also be of interest.

*We thank the reviewer for pointing out these references, and we had added them to the text.*

• The paper needs a table of descriptive statistics – a reader should be able to look at this “Table 1” and easily understand the prevalence and distribution of the key treatment, outcome, and covariates.

*Now added*

• The outcome measures are interesting and (particularly in the questions on gendered harassment) innovative, but vulnerable to social desirability bias (and potentially in either direction – if boys are willing to engage in harassing behavior while out with peers but unwilling to report willingness to engage in this behavior to adults… or if they overreport such willingness in attempts to look tough.). What is the risk that this social desirability is driving your results? What direction would the over/underreporting need to be in, and among whom? Is this plausible?

*The reviewer is of course right that social desirability may well be an issue here. One defence is that significant number of respondents did in fact indicate that it was at least sometimes OK to engage in the behaviours used to construct the social norms scales – there was variation in views. But another point is that social desirability is, plainly, related to what respondents perceive to be dominant social norms. If those high in trust in police, for example, were more motivated to provide what they felt was the ‘right’ answer than those who were lower in trust, this does not undermine our argument – indeed in a sense it is our argument. The issue then becomes the extent to which these expressed opinions are related to more deeply held attitudes, and the extent to which all this guides behaviour. We hope that future work will address these important questions.*

• The structural equation models seem reasonable, though I’m not an expert on this particular modeling method – I imagine other reviewers may be better able to comment on the specifics. That said, I have some concerns about the inferences the authors are making – they are careful to caveat their findings and note that their ability to make causal inferences from cross-sectional data is limited. Nonetheless, the discussion, while well written, clearly suggests causal effects. The authors should be a bit more careful not to overstate their conclusions.

*We have been through the discussion to ensure more careful language is used throughout.*

**Reviewer #2**

• At some points throughout the manuscript, it seems that the findings from Epp et al. (2014) [Pulled Over: How Police Stops Define Race and Citizenship] may have some implications worth noting—though Epp et al.’s research is based in the US. Specifically, the authors look at traffic stops: the type stops the officers make, how the officers interact with the person stopped, the race of the driver stopped. When stops are common, repeated, “unjust,” the process is seen as unfair and conveys messages about equality and citizenship.
o This Epp et al. research came to mind regarding the sentences on line 58 & 59 (page 3) and “Unfair treatment…” on line 221 (page 9)

*We have added this reference*

• Line 86 (page 4) – you say, “mainstream norms of respect and equality between males and females”, but are those the mainstream gender norms? Could power differentials/“traditional” male/female social role divides be understood as more “mainstream”?

*We are grateful for this point. We have removed the note about these being “mainstream” norms. This claim is both unnecessary to our argument and begs questions.*

• I agree that masculinity is an important concept here, but you do not bring up masculinity until page 12. I think it may be worth mentioning earlier. It seems to take a while for the gendered component of your argument to be brought to light though it is central to your study. I would recommend adding more detail at earlier points. For one example, you could maybe add a little more detail when you say: “internalise unhealthy gendered norms” (line 191, page 8).

*We have added references to gender at the place the reviewer suggests, and also earlier in the paper (e.g. line 59 [note that line numbers will have changed with editing). In particular, we have added the following to the introduction:*

“Gendered social norms define the practices that are expected of males and females, typically in binary, heteronormative ways (Burrell et al., 2019). Starting from the premise that certain stereotypical forms of hegemonic masculinities coalesce around notions of toughness, competition and self-protection, as well as sexual objectification in public space and control within intimate-relationships, we consider the idea that gendered norms (based on gendered dominance that render it acceptable to sexually harass in public space and to control romantic partners) could be activated and/or strengthened when normative constraints are loosened by the (subjective) experience of procedural injustice.”

o Do you think the gender of the involved officers matter? What about if there are/are not any witnesses of the S&S experience?

*These are important points, but ones we unfortunately cannot cover in the current paper. We have added a sentence to the limitations section to make the point that ideally these issues would be covered, and should be in future work.*

• Is the additional analysis inclusive of all boys, girls, and those who didn’t answer since the sample size is 1,666? At first it sounded like you were only going to look at girls and those who didn’t answer the gender question here.

*The additional analysis contains the full Year 10/11 sample, i.e. boys, girls, those with another gender identity and those who did not answer the question (note also tht we have corrected the sample size here to 1,752). This seems preferable to simply focussing on girls, primarily because it allows inclusion of the relatively large number of respondents who did not report their gender and whom, therefore, could conceivably be different to other respondents in important ways (e.g. trust). It may be important that in this second model the paths from trust in the police to Norms1 and Norms2 strengthen, and both are significant at the .05 level*

o I am also wondering about your inclusion of cases where individuals didn’t answer the gender question. Does the inclusion of respondents who didn’t answer the gender question make the results less comparable to the main analysis? Why not keep the additional analysis to just girls while the main models look at boys?

*As noted above, it seems more important to include the gender ‘non-responders’ than simply focus on girls. We could of course introduce a third model that looks only those who identified as girls, but this only a small number with stop and search experiences.*

o You say the findings from the additional analysis align closely with the full models, but I’m wondering about possible bias in the results? (--especially if boys were still included here too). And you said 23% of 10 and 11 school year students didn’t answer (about 383 students if my calculation is correct), which is a pretty large percentage.

*See response above.*

• What was the ethnic breakdown of this survey? You control for it, but I’d be interested in seeing what the distribution looks like. Also, what does BME stand for?

*See Table 1 in the new manuscript. BME is a term widely used in the UK that refers to ‘Black and Minority Ethnic’ group (essentially, the non-White part of the population). It is not necessarily our preferred terminology, though, so we have changed to White/non-White in the new manuscript.*

Suggested grammatic and structural edits:

• MOPAC first appears in line 94 (page 5) and should be written out long form at that point, then abbreviated after that.

*Amended*

• The two sentences spanning lines 101-103 (page 5) “It is important to note… our analysis is therefore speculative.”) can probably be removed here and just be included in the limitations section of the Discussion.

*Here we disagree with the reviewer – we think it’s important to signal upfront in the paper that our primary concern is theory-building.*

• The sentence on lines 303-305 sounds a bit awkward (page 12) (“…Lauger (2014) found that…”)

*Amended*

• The sentence spanning line 333-336 (p. 13) (“Capturing gendered normative attitudes…) seems a bit out of place; it can probably wait until the Methods section when you talk about the data and measures used in greater detail.

*Again we respectfully disagree with the reviewer, in as much as mentioning the survey items helps set up the second half of the paragraph, which covers the distinction between positive and negative social (and gendered) norms.*
