# Supplementary material for: A street corner education: Stop and search, trust, and gendered norms among adolescent males
Source: PLoS One. 2022 Dec 27;17(12):e0279505. doi: 10.1371/journal.pone.0279505 (PMC9794084; doi:10.1371/journal.pone.0279505)
Supplement: Attachment — Submitted filename: Response to reviewers.docx [file pone.0279505.s003.docx]

**A street corner education: Stop and search, trust, and gendered norms among adolescent males**

We thank the reviewers for their very positive responses to the revised version of our article, and we are glad they feel it is in the position to make “an important and original contribution to the field” (R2). While R2 had no further comments to make, R1 did raise two final points, which we address below.

*Line 103: I have to agree with my co-reviewer in not liking the language of “our analysis is therefore speculative.” Moreover, I think it sells the paper and it’s contributions short. Maybe refer to the paper as opening, rather than concluding a conversation? Refer to the findings as “generative”? This might be a case of subtle language differences between the US and the UK, but my read of “speculative” in this case is “not well grounded”, and I don’t think that’s the case with this paper.\*

We thank the reviewer for this comment, which is actually very positive, and we have amended the text at Line 103 along the lines they suggest: “our analysis should therefore be considered generative in nature”.

*Line 491: I appreciate the inclusion of table 1. However, can you split the true “no” answers on police contact from the “don’t know” responses, even if you need to combine them for your models? I find this breakdown itself to provide some insight into the kids’ legalization (I.e., how well they understand crime and police contact and how willing they are to discuss it.)*

We have added the ‘don’t know’ (n=19) and ‘did not answer’ (n=2) responses to the stop and search question, and ‘don’t know’ (n=50) and ‘don’t want to say’ (n=36) responses to Table 1 on p.19. Note this means footnotes to the table are no longer required.
